# Caesarean section audit to improve quality of care in a rural referral hospital in Tanzania

**DOI:** 10.1186/s12884-018-1814-1

**Published:** 2018-05-15

**Authors:** Luuk Dekker, Tessa Houtzager, Omary Kilume, John Horogo, Jos van Roosmalen, Angelo Sadock Nyamtema

**Affiliations:** 1 Department of Obstetrics and Gynaecology, St. Francis Referral Hospital, Ifakara, Tanzania; 2 Department of Obstetrics, Leiden University Medical Centre, Leiden, The Netherlands; 3 St. Francis University College of Health and Allied Sciences, P.O Box 175, Ifakara, Tanzania

**Keywords:** Caesarean section, Assisted vaginal delivery, Vacuum delivery, Audit, Tanzania, Decision-to-delivery interval

## Abstract

**Background:**

Caesarean section (CS) is often a life-saving procedure, but can also lead to serious complications, even more so in low-resource settings. Therefore unnecessary CS should be avoided and optimal circumstances for vaginal delivery should be created. In this study, we aim to audit indications for Caesarean sections and improve decision-making and obstetric management.

**Methods:**

Audit of all cases of CS performed from January to August 2013 was performed in a rural referral hospital in Tanzania. The study period was divided in three audit blocks; retrospective (before auditing), prospective 1 and prospective 2. A local audit panel (LP) and an external auditor (EA) judged if obstetric management was adequate and indications were appropriate or if CS could have been prevented and yet retain good pregnancy outcome. Furthermore, changes in modes of deliveries, overall pregnancy outcome and decision-to-delivery interval were monitored.

**Results:**

During the study period there were 1868 deliveries. Of these, 403 (21.6%) were Caesarean sections. The proportions of unjustified CS prior to introduction of audit were as high as 34 and 75%, according to the respective judgments of LP and EA. Following introduction of audit, the proportions of unjustified CS decreased to 23% (*p* = 0.29) and 52% (*p* = 0.01) according to LP and EA respectively. However, CS rate did not change (20.2 to 21.7%), assisted vacuum delivery rate did not increase (3.9 to 1.8%) and median decision-to-delivery interval was 83 min (range 10 - 390 min).

**Conclusions:**

Although this is a single center study, these findings suggest that unnecessary Caesarean sections exist at an alarming rate even in referral hospitals and suggest that a vast number can be averted by introducing a focused CS audit system. Our findings indicate that CS audit is a useful tool and, if well implemented, can enhance rational use of resources, improve decision-making and harmonise practice among care providers.

## Background

Caesarean section (CS) is a lifesaving procedure when spontaneous or assisted vaginal delivery is not possible. The World Health Organization (WHO) recommends a rate of 5 – 15% for any community and above that is considered unnecessary overuse of the procedure. In Tanzania, the population-based CS-rate in 2010 was 4.5% and in rural areas only 3.2%, indicating general underuse [1, 2]. However, in some health facilities CS-rates are much higher and probably overused.

Although CS in general is a safe operation, the procedure can lead to serious complications. These include endometritis, wound haematoma and infection, venous thromboembolism, anaesthetic complications, infertility and abdominal adhesions which can lead to chronic abdominal and pelvic pain as well as a risk of injury to adjacent organs in future surgeries [3–8]. Furthermore, CS bears consequences for subsequent pregnancies, with higher risks of excessive blood loss, uterine scar rupture, placenta accreta, placenta praevia and abruptio placentae [9–12]. Most of these complications are more serious in resource-limited settings, reinforcing the restraint which should be used in deciding to perform CS.

In addition to these medical complications, CS is associated with considerable costs for patients and hospitals, resulting in a longer hospital stay, whilst the number of available beds in most centres is limited. Therefore, to avoid unnecessary CS, management of women in labour should be appropriate and the decision for CS be made only in situations where no better alternatives are available [13].

Few audits measuring adequacy of decision-making for CS have formally been evaluated in countries such as Tanzania [14–17]. In an attempt to improve health care and reduce maternal and perinatal mortality and morbidity, audit was introduced in St. Francis Referral Hospital (SFRH) in Ifakara, Tanzania [18]. The research questions in this study were: ‘What is the current magnitude of unnecessary CS at SFRH?’ ‘Can CS audit be used to reduce unnecessary CS?’ ‘Can CS audit lead to harmonisation of decision-making, enhance alternative delivery modalities and improve maternal care?’ To answer these questions, we established a CS audit system at SFRH, hoping that if successful the initiative may provide an appropriate template for the future use of an audit-based system to reduce unnecessary CS and enhance alternative modes of deliveries in resource-poor settings.

## Methods

The study was conducted in St. Francis Referral Hospital, a referral hospital located in Ifakara, headquarters of Kilombero district in Morogoro region in south-western Tanzania. The district covers an area of 14,918 km^2^ with a population of 407,880 as per 2012 population census. Seventy-two percent of inhabitants live in rural areas, 81% of the population being farmers [19]. SFRH has 372 beds and an average annual delivery rate of 5200 [18]. Although prenatal services are provided at all levels of health care including dispensaries, patients requiring higher level of care may have to walk up to 75 km to get to SFRH. In view of this, SFRH also provides care to patients from the neighbouring districts (Ulanga, Kilosa and Morogoro rural districts).

Cases of CS were collected retrospectively up to 7 weeks from January until February and prospectively in two blocks from February until August, 2013. For data-collection, the same sources were used for the retro- and prospective periods, being case files, partographs, antenatal-care cards and delivery records books. Data items collected included gravidity, parity, modes of previous deliveries including the number of previous Caesarean sections, gestational age, time at decision for CS and time at start of surgery.

The local investigators summarised all cases of CS and presented them anonymously to a local audit panel (LP) using powerpoint-presentations. This local panel included all obstetricians, intern doctors, assistant medical officers, medical students and midwives present at the time of clinical morning discussions. Involvement of all doctors was encouraged in order to improve knowledge and harmonise obstetric practices in the department. They critically discussed the cases and reached consensus if obstetric management was appropriate and indications were conform the hospital standards or if CS could have been prevented. The investigators were present during presentations and the LP was able to ask them for extra information if needed before making its final judgment. Furthermore, an external auditor (EA) evaluated the cases as well, since it is known that external and internal analysis can differ significantly [20]. The EA was a senior obstetrician who was familiar with the resources available and the circumstances in which obstetric care is provided in SFRH. The same presentations, complemented with additional information if requested, were sent to the EA for second judgment. These judgments were based on, among others, the National CEmOC management guidelines, WHO recommendations and other nationally recommended reference books [21, 22]. Unjustified indications for an emergency CS were for example: ‘prolonged/obstructed labour’ with intact membranes, ‘prolonged/obstructed labour’ in primigravidae without oxytocin augmentation, ‘prolonged/obstructed labour’ with descent ≤3/5 (level of the Ischial Spine, station 0) without a trial of vacuum extraction or ‘prolonged/obstructed labour’ before crossing of the action line. If CS was judged to be unjustified, this did not mean CS could have definitely been prevented, but it implies that optimal circumstances for vaginal delivery were not created and that CS might have been avoided.

Main outcome measures were: Modes of deliveries, indications for CS, judgment on obstetric management and justification of CS by local audit panel and external auditor, maternal morbidity and mortality and decision-to-delivery interval.

In order to further enhance analysis, indications for CS were classified into groups based on their similarities and agreed management approaches. CS performed in women with ≥2 previous uterine scars were excluded from the analyses, since this indication is always considered to be justified based upon the national guidelines for emergency obstetric care. Asides from these, a classification system of indications was developed because there usually was more than one indication contributing to the decision to perform CS such as ‘Foetal Distress + Prolonged Labour’, ‘Obstructed Labour + inadequate contractions’ or ‘Cephalopelvic Disproportion + Foetal Heart Rate <100 bpm’. In these combinations of indications, no consistency in primary indication could be identified. Moreover, often indications such as prolonged labour and obstructed labour were not distinguished consistently. Lastly, classification in these groups was also done in order to retain enough statistical power to demonstrate clinically important differences.

The three groups of indications were:

- Group 1: failed trial of scar;
- Group 2: cephalopelvic disproportion/prolonged or obstructed labour/arrested descent/foetal distress (without previous scars);
- Group 3: other than group 1 & 2.

Group 3 included indications such as malpresentation, (pre)eclampsia, multiple pregnancy, bad obstetric history, cord prolapse, placenta praevia or abruptio placenta.

Judgments of local audit panel and external auditor were documented and analysed. Furthermore, to determine the impact of the audit, proportions of unjustified CS and trends of CS and vacuum delivery rate were compared between the three study blocks. For exploring the quality of health care, maternal outcome was assessed. Morbidity was evaluated using the Haydom-variant of the internationally established Maternal-Near-Miss criteria of the WHO, since these criteria are better suited in low-resource settings like SFRH [23].

All statistical analyses were computed using SPSS version 20 (SPSS, Inc., USA). Differences in modes of deliveries and judgments over the audit periods were analysed using chi-square tests with two-sided *p*-values. Outcomes were considered significant if *p* < 0.05. Case by case correlation was calculated using Cohen’s kappa for agreement between local panel and external auditor. Permission to conduct the study was obtained from the hospital management and ethical clearance as part of the larger audit project in the hospital [17]. After having evaluated all cases locally, a final presentation was held in SFRH with tentative results. These were discussed and recommendations were made for improving future obstetric care.

## Results

The study period covered 179 days which were divided into three audit blocks, one retrospective block followed by two prospective blocks. Over this period, 1868 deliveries took place in SFRH. Mean maternal age was 27 years, mean parity 1.6 and mean gestational age 39 weeks (Table 1).

**Table 1:** Characteristics of women who delivered by Caesarean section during the study period

Basic indicators	Number	Mean (SD)
Maternal age in years		26.6 (6.5)
Gravidity		2.7 (1.6)
1	99	
2	75	
3+	160	
Parity		1.6 (1.6)
0	101	
1	80	
2+	153	
Number of living children		1.3 (1.4)
0	120	
1	87	
2+	120	
(unknown)	(7)	
Gestational age in weeks		38.8 (2.8)
HIV-positive status	20 (10.2%)	
Maternal-near-miss	26 (7.8%)	
Maternal deaths	5 (1.5%)	

The table provides a statistical description of the population of women who were included in this study and an overview of the maternal morbidity (defined as maternal-near-misses) and mortality

Out of all deliveries, 403 (21.6%) were by Caesarean section and 44 (2.4%) were assisted by vacuum (Table 2). Of all cases of CS, 334 (82.9%) were summarised and discussed. Cases were not analysed either because files from the retrospective block could not be retrieved or due to other logistical reasons in the prospective blocks, most often being the investigator temporarily not being present at SFRH for a limited period of time. Out of these 334 cases, 99 (30%) were primigravidae and 156 (47%) had at least one previous uterine scar. There were 26 maternal-near-misses, almost all of them due to eclampsia, ≥2 units of blood, uterine rupture or a combination of these. Furthermore, five (1.5%) maternal deaths occurred. Two of these were due to complications of abruptio placentae, one was due to eclampsia, one due to uterine rupture and one due to postpartum haemorrhage. In four of five maternal deaths (80%) CS was found unjustified. All CS in six cases of uterine rupture were justified, however one CS was found to be performed too late. Over the three audit blocks there were no significant changes in modes of deliveries.

**Table 2:** Overtime trends of proportions of modes of deliveries and decision-to-delivery interval

Delivery indicators	Retrospective block *n* (%)	Prospective block 1 *n* (%)	Prospective block 2 *n* (%)	Total *n* (%)	Chi-square test for differences in mode of delivery across the blocks
Audit period in days	51	39	89	179	
Total deliveries	415 (100)	429 (100)	1024 (100)	1868 (100)	
Vaginal deliveries	315 (75.9)	322 (75.1)	784 (76.6)	1421 (76.1)	*p* = 0.83 (χ^2^=0.4)
Caesarean sections	84 (20.2)	97 (22.6)	222 (21.7)	403 (21.6)	*p* = 0.70 (χ^2^=0.7)
Vacuum deliveries	16 (3.9)	10 (2.3)	18 (1.8)	44 (2.4)	*p* = *0.06* (χ^2^=5.7)
Caesarean sections analysed	70 (83.3)	96 (99.0)	168 (75.7)	334 (82.9)	
Decision-to-delivery interval for emergency CS in minutes; median (range)	75 (15-340)	60 (15-390)	95 (10-365)	83 (10-390)	

The table shows a numerical overview of the blocks, including the number of cases collected, modes of deliveries (with chi-square tests for differences in modes of deliveries over the blocks) and decision-to-delivery intervals per block

Mean APGAR at 1 min was 8.0 and after 5 min was 9.5, two neonates having an APGAR < 4 after 5 min. Of 190 neonates of which outcome is known, 21 (11.1%) were admitted to the neonatal ward. Asides, six neonates (3.2%), of which one pair were twins, died after CS. Of these five cases of CS, two were judged as unjustified. There were seven cases of intra-uterine fetal death (3.7%), of which in five CS was unjustified. None of the adverse pregnancy outcomes was directly attributed to the CS procedure.

Of the 334 analysed cases, 64 CS (19%) were performed due to a history of two or more previous uterine scars and these were excluded from further analysis. Of the remaining 270 cases over all three blocks in total, LP judged 70 (26%) and EA judged 157 (58%) CS to be unjustified. Cohen’s kappa for case by case correlation was 0.27, suggesting fair agreement between LP and EA.

**Fig. 1:**
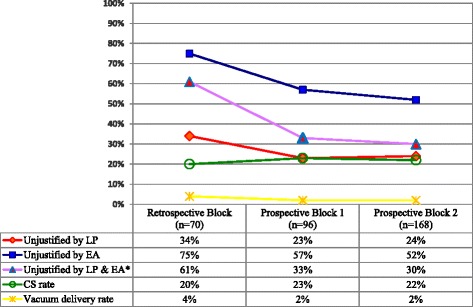
Overtime trends of the proportions of unjustified Caesarean sections and modes of deliveries. Content: A multiple line graph with concomitant table displaying the trends of the percentages of unjustified CS over the blocks as judged by the local audit panel and the external auditor, as well as the trends of modes of deliveries (CS-rate and vacuum delivery rate). *Abbreviations: SFRH = St. Francis Referral Hospital; LP = local audit panel; EA = external auditor; CS = Caesarean section*

Of retrospectively analysed cases, proportions of unjustified CS were 34% according to the local audit panel and 75% according to the external auditor. Following introduction of audit, this dropped to 23% for LP (*p* = 0.29) and 52% for EA (*p* = 0.01) (Fig. 1). In view of the different groups of indications, the most notable declines of unjustified CS, especially according to the local panel, were seen in group 1 [trial of scar] and 2 [cephalopelvic disproportion/prolonged or obstructed labour/arrested descent/foetal distress] (Table 3). The major reasons for persistent unjustified CS in these groups were failure to adhere to the principles of trial of labour or trial of scar. These included the presence of intact membranes at time of decision for emergency CS, absence of oxytocin augmentation in case of inadequate contractions or not attempting to perform vacuum delivery prior to emergency CS when the cervix was fully dilated and descent ≤3/5. Almost a quarter (24%) of 152 women who had emergency CS after a failed trial of labour or scar (group 1 or 2) still had intact membranes when CS was performed and 28 (18%) reached full dilatation and a descent of ≤3/5, indicating that they could have had a trial of assisted vacuum delivery. Of all 152 cases of emergency CS, two women had a failed trial of vacuum prior to CS.

**Table 3:** Overtime trends of proportions of unjustified Caesarean sections per group of indications

Groups of indications for CS	Retrospective Block		Prospective Block 1		Prospective Block 2		Chi-square test for differences across the blocks
	Total *n*	Unjustified *n* (%)	Total *n*	Unjustified *n* (%)	Total *n*	Unjustified *n* (%)	
Group 1 [Failure of trial of scar]							
Judgment by LP	10	3 (30%)	15	3 (20%)	20	4 (20%)	*p* = 0.80 (χ^2^=0.5)
Judgment by EA	10	8 (80%)	15	7 (47%)	20	12 (60%)	*p* = 0.25 (χ^2^=2.8)
Group 2 [Cephalopelvic disproportion/obstructed or prolonged labour/arrested descent/foetal distress]							
Judgment by LP	27	14 (52%)	24	6 (25%)	56	13 (23%)	*p* = 0.24 (χ^2^=7.5)
Judgment by EA	27	23 (85%)	24	14 (58%)	56	34 (61%)	p *= 0.06* (χ^2^=5.8)
Group 3 [other than group 1&2]							
Judgment by LP	22	3 (14%)	38	9 (24%)	58	15 (26%)	*p* = 0.50 (χ^2^=1.4)
Judgment by EA	22	13 (59%)	38	23 (61%)	58	23 (40%)	*p = 0.09* (χ^2^=4.9)

The table shows a numerical overview of the subgroups by indications per block, including the number of unjustified CS within those, with chi-square tests for differences in justification over the blocks

*Abbreviations*: *LP* local audit panel, *EA* external auditor, *CS* Caesarean section

After introduction of audit, Caesarean section rate did not change (20.2 to 21.7%) and assisted vacuum delivery rate did not increase (3.9 to 1.8%). However, it was noted that assisted vacuum delivery was registered by midwives in the delivery book as spontaneous vaginal delivery quite often. Because of incomplete documentation only 215 emergency CS cases could be assessed for the time interval from decision for emergency CS to the start of surgery. The median interval was 83 min (range 10 – 390). Only one third (33%) got an emergency CS within an hour after decision and in 28 cases (13%) CS was performed three hours or more after the decision was made.

## Discussion

Introduction of audit was associated with a reduction of unjustified CS of 11% (from 34 to 23%) and 23% (from 75 to 52%) according to the LP and EA, suggesting audit is a promising tool. This reduction of unjustified CS suggests enhancement of knowledge and harmonisation of decision-making among care providers, indicating improved quality of management of labour.

However, this study also has limitations. Firstly, this was a single center study, although SFRH is quite representative for rural hospitals in Tanzania. It would be interesting to see whether similar results could be acquired in other hospitals. Secondly, the drop of unjustified CS could be explained by the presence of high proportions of unjustified CS from the start. Thirdly, one fifth of CS could not be analysed due to poor documentation or logistical reasons. However, we found no reason to assume these CS are different from the analysed CS and may have led to bias. Finally, in order to maintain these results CS audit should be regularly done so that knowledge and harmony of the decision-making process is continuously transmitted to new care providers joining the obstetric department.

Globally, there is increasing concern about the rising trends of CS rates. General agreement is that, although Caesarean sections have become a much safer procedure over the years, it cannot replace vaginal delivery in terms of low maternal and neonatal morbidity and less expense [24]. In accordance with other reports, almost half (47%) of women who delivered by CS at SFRH had at least one previous uterine scar [25]. It is known that repeat CS rates are high in Western countries as well, numbers up to 76% having been reported [26]. These findings suggest that primary Caesarean section usually determines the future obstetric course of women and therefore should be avoided if possible. This is probably even more important in the more rural areas with limited access to health care. Again similar to other reports, our study shows that failure to progress and foetal distress were the leading indications for emergency CS, while two or more previous uterine scars was the most common indication for elective CS [27, 28].

The fact that a vast number of CS performed before introduction of audit at SFRH was unjustified suggests an alarming rate of unnecessary CS in this hospital and probably also in other Tanzanian hospitals. This needs urgent attention. Like many other studies from resource-limited countries, unjustified CS are mainly attributed to poor decision-making. Decisions for CS are often made by junior care providers who are less trained and get little supervision [29]. The LP acknowledged the difficulty not to rely too much on intern doctors, since senior doctors are not always present. They agreed that decision-making for CS requires more experience and knowledge and should be discussed more extensively with midwives and senior doctors. There was long-existing peer review by the obstetric department team of the Caesarean sections performed in the preceding 24 h during clinical morning discussions. This practice, however, did not seem to improve decision-making or harmonise practice on CS. This suggests a need to restructure clinical morning discussions to enhance decision-making and improve quality of care.

The difference in judgements between local audit panel and external auditor was notable. Although cases were submitted anonymously and the majority of staff would not have been involved in the management of most patients, the LP was judging their own actions, perhaps causing a less critical approach of the cases than the EA. Since the EA analysed all patients on his own and was not present during the panel meetings, he might have interpreted available information differently. Furthermore, since the EA is particularly involved in reducing unnecessary interventions he could be biased against actual appropriate indications.

Although no significant changes in trends of proportions of unjustified CS per group were found, probably due to the small number of cases per group per block, a decrease of unjustified CS was seen in all different groups of indications after introduction of the audit. The LP itself also thought the audit to be very useful and was positive about its effects, especially regarding its function as a “learning platform”. Considering the impact found in this study, introduction of CS audit is recommended in all hospitals in Tanzania and other resource-limited countries.

Unexpectedly, despite the decrease in unjustified CS, Caesarean section rate nor assisted vacuum delivery rate changed significantly (20.2 to 21.7% and 3.9 to 1.8% respectively). A possible explanation could be that a group of women existed who needed CS, but did not receive it before the start of this audit. During the course of the audit, this group may have been acknowledged as suitable candidates for CS, while a group of women in whom CS was not indicated did not get the operation. This is subject for further study. Part of the low rate of assisted vacuum deliveries could be attributed to incorrect documentation, as investigators noticed that deliveries by vacuum were sometimes recorded by midwives as spontaneous vaginal deliveries. However, considering that assisted vacuum delivery could have been attempted in 18% of women from group 1 and 2 and was attempted in only two cases prior to emergency CS, it is likely that it is currently not used to its full potential. Operative vaginal delivery is known to be safe and contributes to more than 10% of deliveries in most European and Northern American countries [30]. All together, these findings prompt a need to promote the use of vacuum delivery, as it may also contribute to further reduction of unnecessary CS. During the final discussion, the LP recognised this issue and explained that vacuum extractions fell from grace because of a possibly increased risk of HIV-infection for the foetus. However, they agreed that no convincing scientific data was available to support this and that too few vacuum deliveries were performed in HIV-negative women as well, although they make up almost 90% of population.

Considering the generally accepted period of time between decision for and start of emergency CS is 60 min at most, the median decision-to-delivery interval of 83 min was striking. Furthermore, 13% were performed more than three hours after decision for emergency CS, while it is known that a decision-to-delivery interval of more than 75 min is already associated with poorer maternal and neonatal outcome [31]. This delay may be caused by the amount of time that elapses for haemoglobin and blood group analysis and preparation of the operating theatre, although there are two other operating theatres that could also be used in case of emergencies. Also, the limited number of available staff may contribute to this delay. However, the LP realised that a necessity for a reduction of the decision-to-delivery interval was obvious and stated that another study was needed to investigate the causes of this delay and to assess methods for improvement of the current situation.

## Conclusions

Even though this was a relatively small study performed in one center, our findings suggest that unjustified Caesarean sections exist at alarming rates, even in referral hospitals, and suggest that a vast part can be averted by introducing a focused CS audit system. Our study findings suggest that CS audit is a very useful tool and, if well implemented, can improve decision-making and harmonise practice among care providers. Furthermore, decision-to-delivery interval should be reduced and monitored and the use of vacuum delivery should be emphasised. In view of these results CS audit is strongly recommended in all hospitals in resource-limited countries.

## Abbreviations

CS: Caesarean section
EA: External auditor
LP: Local (audit) panel
SFRH: St. Francis Referral Hospital
WHO: World Health Organization

## References

[1] Cavallaro FL, Cresswell JA, Franca GV, Victora CG, Barros AJ, Ronsmans C (2013). Trends in caesarean delivery by country and wealth quintile: cross-sectional surveys in southern Asia and sub-Saharan Africa. Bull World Health Organ.

[2] National Bureau of Statistics. United Republic of Tanzania demographic and health survey 2010. Tanzania: Dar-Es-Salaam; 2011.

[3] Burrows LJ, Meyn LA, Weber AM (2004). Maternal morbidity associated with vaginal versus cesarean delivery. Obstet Gynecol.

[4] Liu S, Liston RM, Joseph KS, Heaman M, Sauve R, Kramer MS (2007). Maternal mortality and severe morbidity associated with low-risk planned cesarean delivery versus planned vaginal delivery at term. CMAJ.

[5] Jacobsen AF, Skjeldestad FE, Sandset PM (2008). Incidence and risk patterns of venous thromboembolism in pregnancy and puerperium--a register-based case-control study. Am J Obstet Gynecol.

[6] Murphy DJ, Stirrat GM, Heron J, Team AS (2002). The relationship between caesarean section and subfertility in a population-based sample of 14.541 pregnancies. Hum Reprod.

[7] Nikolajsen L, Sorensen HC, Jensen TS, Kehlet H (2004). Chronic pain following caesarean section. Acta Anaesthesiol Scand.

[8] Nisenblat V, Barak S, Griness OB, Degani S, Ohel G, Gonen R (2006). Maternal complications associated with multiple cesarean deliveries. Obstet Gynecol.

[9] Daltveit AK, Tollanes MC, Pihlstrom H, Irgens LM (2008). Cesarean delivery and subsequent pregnancies. Obstet Gynecol.

[10] Usta IM, Hobeika EM, Musa AA, Gabriel GE, Nassar AH (2005). Placenta previa-accreta: risk factors and complications. Am J Obstet Gynecol.

[11] Yang Q, Wen S, Oppenheimer L, Chen X, Black D, Gao J (2007). Association of caesarean delivery for first birth with placenta praevia and placental abruption in second pregnancy. BJOG.

[12] Gurol-Urganci I, Cromwell DA, Edozien LC, Smith GC, Onwere C, Mahmood TA (2011). Risk of placenta previa in second birth after first birth cesarean section: a population-based study and meta-analysis. BMC Pregnancy Childbirth..

[13] Souza JP, Gulmezoglu A, Lumbiganon P, Laopaiboon M, Carroli G, Fawole B (2010). Caesarean section without medical indications is associated with an increased risk of adverse short-term maternal outcomes: the 2004-2008 WHO global survey on maternal and perinatal health. BMC Med.

[14] Maaløe N, Bygbjerg IC, Onesmo R, Secher NJ, Sorensen BL (2012). Disclosing doubtful indications for emergency cesarean sections in rural hospitals in Tanzania: a retrospective criterion-based audit. Acta Obstet Gynecol Scand.

[15] Maaløe N, Sorensen BL, Onesmo R, Secher NJ, Bygbjerg IC (2012). Prolonged labour as indication for emergency caesarean section: a quality assurance analysis by criterion-based audit at two Tanzanian rural hospitals. BJOG.

[16] Heemelaar S, Nelissen E, Mdoe P, Kidanto H, van Roosmalen J, Stekelenburg J (2016). Criteria-based audit of caesarean section in a referral hospital in rural Tanzania. Tropical Med Int Health.

[17] Nyamtema AS, de Jong AB, Urassa DP, van Roosmalen J (2011). Using audit to enhance quality of maternity care in resource limited countries: lessons learnt from rural Tanzania. BMC Pregnancy Childbirth.

[18] St. Francis Referral Hospital website. Available online at: http://www.ifakara.org/en/st-francis-hospital/hospital.php (accessed August 10th, 2015).

[19] National Bureau of Statistics. United Republic of Tanzania population and housing census 2012. Tanzania: Dar-Es-Salaam; 2013.

[20] Sorensen BL, Elsass P, Nielsen BB, Massawe S, Nyakina J, Rasch V (2010). Substandard emergency obstetric care - a confidential enquiry into maternal deaths at a regional hospital in Tanzania. Tropical Med Int Health.

[21] WHO, UNFPA, UNCEF, World Bank. Integrated Management of Pregnancy and Childbirth. Managing Complications in Pregnancy and Childbirth: a guide for doctors and midwives. WHO; 2000.

[22] Cunningham F, Leveno K, Bloom S, Hauth J, Gilstrap L, Wenstrom K (2005). Williams obstetrics.

[23] Nelissen E, Mduma E, Broerse J, Ersdal H, Evjen-Olsen B, van Roosmalen J (2013). Applicability of the WHO maternal near miss criteria in a low-resource setting. PLoS One.

[24] Sachs BP, Kobelin C, Castro MA, Frigoletto F (1999). The risks of lowering the cesarean-delivery rate. N Engl J Med.

[25] Wareham V, Bain C, Cruickshank D (1993). Caesarean section audit by peer review. Eur J Obstet Gynecol Reprod Biol.

[26] Wray J (2001). Review of the National Sentinel Caesarean Section Audit Report. Pract Midwife.

[27] CPY R. A clinical audit on caesarean section: indications and outcomes [PhD thesis]. Hong Kong: University of Hong Kong; 2003.

[28] Naidoo N, Moodley J (2009). Rising rates of caesarean sections: an audit of caesarean sections in a specialist private practice. SA Fam Pract.

[29] Ion R, Alatt H (2013). Reducing the caesarean section rate in a rural hospital in south-West Uganda. Arch Dis Child Fetal Neonatal Ed.

[30] Thomas J, Paranjothy S (2001). Royal College of Obstetricians and Gynaecologists clinical effectiveness support unit: National Sentinel Caesarean Section Audit Report.

[31] Thomas J, Paranjothy S, James D (2004). National cross sectional survey to determine whether the decision to delivery interval is critical in emergency caesarean section. BMJ.

